# Ganglion Cell-Inner Plexiform Layer, Peripapillary Retinal Nerve Fiber Layer, and Macular Thickness in Eyes with Myopic *β*-Zone Parapapillary Atrophy

**DOI:** 10.1155/2016/3746791

**Published:** 2016-10-27

**Authors:** Jin-woo Kwon, Jin A. Choi, Jung-sub Kim, Tae Yoon La

**Affiliations:** ^1^Department of Ophthalmology and Visual Science, St. Vincent's Hospital, College of Medicine, Catholic University of Korea, Seoul, Republic of Korea; ^2^B & VIIT Eye Center, Seoul, Republic of Korea

## Abstract

*Purpose*. To assess the correlations of myopic *β*-zone parapapillary atrophy (*β*-PPA) with the optic nerve head (ONH) and retina.* Methods*. We selected 27 myopic patients who showed prominent *β*-PPA in one eye and no *β*-PPA in the other eye. We studied their macula, macular ganglion cell-inner plexiform layer (mGCIPL), peripapillary retinal nerve fiber layer (pRNFL) thickness, and ONH parameters using optical coherence tomography.* Results*. The average of five out of six sectors and minimum values of mGCIPL thicknesses in eyes with prominent *β*-PPA discs were significantly less than those of the control eyes. The results of clock-hour sector analyses showed significant differences for pRNFL thickness in one sector. In the ONH analyses, no significant difference was observed between myopic *β*-PPA and control eyes. The macular thickness of the *β*-PPA eyes was thinner than control eyes in all sectors. There was a significant difference between the two groups in three sectors (the inner superior macula, inner temporal macula, and inner inferior macula) but there was no significant difference in the other sectors, including the fovea.* Conclusions*. The myopic *β*-PPA eyes showed thinner mGCIPL, parafovea, and partial pRNFL layers compared with myopic eyes without *β*-PPA.

## 1. Introduction

Myopia is one of the most common ocular disorders in the world [1], and the myopic population has been growing significantly in Southeast Asia in recent years [2–6]. The costs of examinations and surgical corrections of myopia are significant, and this disorder has been associated with other pathological eye conditions, such as macular and retinal degeneration, foveoschisis, and rhegmatogenous retinal detachment [7–9]. In addition, studies have reported an association of glaucoma and myopia [10–13], but the mechanism involving how myopia increases the risk of glaucoma is still unknown. The temporal myopic crescent, also known as the *β*-zone parapapillary atrophy (*β*-PPA), is a white, well-defined boundary area with visible sclera due to uncovering of the retinal pigment epithelium, located temporal to the optic disc, which occurs in about 66% of myopic eyes [14–17]. With the recent development of optical coherence tomography (OCT), some studies of *β*-PPA define its area as between the end of Bruch's membrane and the beginning of the retinal pigment epithelium [18, 19].

This tilted change of the disc in myopic eyes can lead to erroneous diagnoses of glaucoma in patients [15, 20] and can also be a risk factor for glaucoma [21]. Optic disc torsion in myopia can also lead to unilateral glaucomatous-appearing visual field (VF) defects [22]. However, the effects of *β*-PPA on glaucoma and retinal degeneration are still unclear [19, 23–25]. To assess the correlations of *β*-PPA with the disc and retina, we selected myopic patients who showed prominent *β*-PPA in one eye and no *β*-PPA in the other eye. We analyzed their macula, macular ganglion cell-inner plexiform layer (mGCIPL), peripapillary retinal nerve fiber layer (pRNFL) thickness, and optic nerve head (ONH) parameters.

## 2. Methods

The medical records of all patients with myopia, defined as a spherical equivalent (SE) ≤ −0.5 diopters (D), who underwent preoperative examination for refractive surgery (laser in situ keratomileusis [LASIK] or surface ablation, including laser epithelial keratomileusis [LASEK], epi-LASIK, or phakic intraocular lens insertion) at the B & VIIT Eye Center, Seoul, Republic of Korea, were reviewed retrospectively. This study was performed according to the tenets of the Declaration of Helsinki, and the study protocol was approved by the institutional review/ethics boards of the Catholic University of Korea, St. Vincent's Hospital, Suwon. Informed consent was not obtained because this study was performed by chart review and the patients' records and information were anonymized and deidentified prior to the analyses.

All patients underwent a full ophthalmological examination that included measuring the visual acuity (VA) and refraction, measuring the intraocular pressure (IOP) using Goldmann applanation tonometry, a dilated fundus examination, stereo disc photometry, and retinal photography using a digital retina camera (CR-1 Mark II; Cannon, Tokyo, Japan) after maximum pupil dilatation and standard perimetry (24-2 Swedish interactive threshold algorithm,* standard automated perimetry*, Humphrey Field Analyzer II; Carl Zeiss Meditec, Dublin, CA, USA) and optical coherence tomography (OCT) (Cirrus High Definition-OCT; Carl Zeiss Meditec, Dublin, CA, USA).

**Figure 1:**
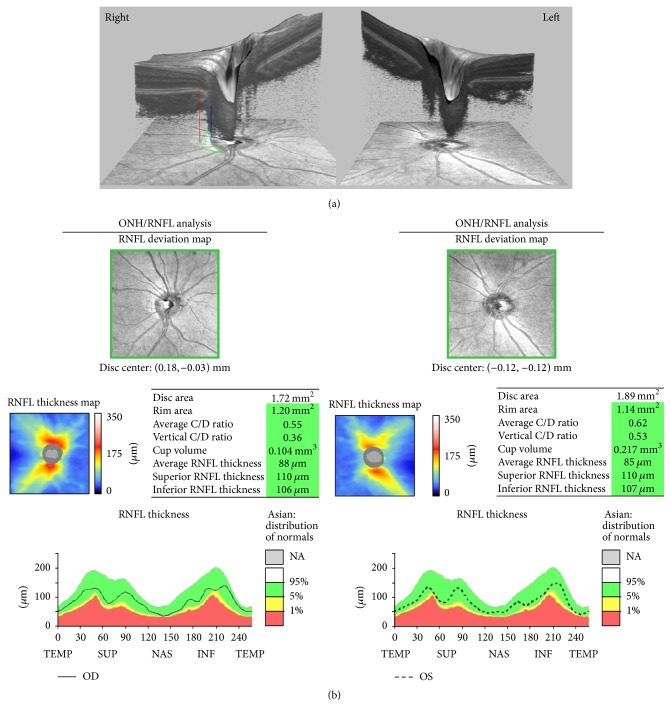
The optical coherence tomography (OCT) and optic nerve head (ONH) image of a 19-year-old female with prominent *β*-zone parapapillary atrophy (*β*-PPA) in the right eye and no *β*-PPA in the left eye. The spherical equivalent of refractive error was −4.75 diopters (D) in the right eye and −5.00 D in the left eye. (a) The en face and cross-sectional optic nerve head OCT images show sections of the *β*-PPA area. The red line designates the end of the retinal pigment epithelium, and the margin of the *β*-PPA and the blue line designate the optic disc margin. The area surrounded by the green line is the *β*-PPA. (b) The OCT results of ONH parameters and peripapillary retinal nerve fiber layer thickness.

Inclusion criteria included myopic eyes showing prominent *β*-PPA in one eye and no *β*-PPA in the other eye (Figure 1). Both eyes showed no glaucomatous disc changes (e.g., large cup-to-disc ratios and an acquired pit of the optic nerve), an absence of any glaucomatous VF defects, and no retinal degeneration including staphyloma. We enrolled patients who were under 40 years of age to reduce age-related effects in the retina.

To eliminate eyes with pathological myopia, eyes with SE > 8.0 D of myopia and pathological retinal lesions, such as a lacquer crack or Fuchs' spot, were excluded [26]. Eyes with concurrent diseases other than refractive error with a best-corrected VA < 20/20, an IOP > 21 mmHg in either eye, a history of severe ocular trauma, intraocular or refractive surgery, evidence of diabetes or other vitreoretinal disease in either eye, evidence of optic nerve or RNFL abnormality in either eye, media opacity, or anisometropia > 2 D were excluded [27].

**Figure 2:**
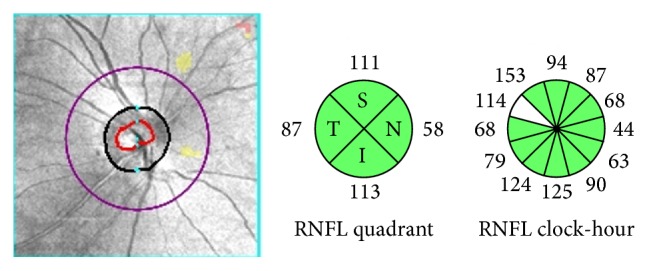
Example of the pRNFL (peripapillary retinal nerve fiber layer) of optical coherence tomography scans showing the area with a radius of 1.73 mm involving the concentric center of the optic disc. The area was divided into four quadrants (superior [S], temporal [T], inferior [I], and nasal [N]) and 12 clockwise sectors of the right eye.

**Figure 3:**
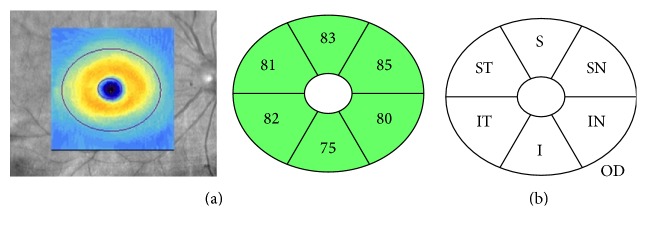
An example (a) and a schematic diagram (b) of an optical coherence tomography scan of the macular ganglion cell-inner plexiform layer, showing the area of the macula with a 4.0-mm-long × 4.8-mm-wide oval shape (excluding a 1.0-mm × 1.2-mm ellipse), centered on the fovea of the right eye. ST, superotemporal; S, superior; SN, superonasal; IN, inferonasal; I, inferior; IT, inferotemporal; OD, right eye.

**Figure 4:**
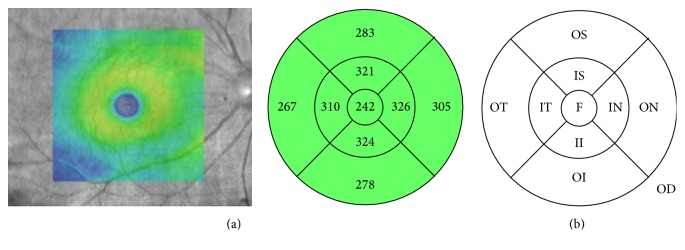
An example (a) and a schematic diagram (b) of a macular optical coherence tomography (OCT) scan showing areas of the fovea with a 1.0-mm concentric diameter, the inner macular area with a 3.0-mm concentric diameter, and the outer macular area with a 6.0-mm concentric diameter. The numbers refer to the average thickness of each macular sector. F, fovea; S, superior; IS, inner superior; OS, outer superior; IN, inner nasal; ON, outer nasal; II, inner inferior; OI, outer inferior; IT, inner temporal; OT, outer temporal; OD, right eye.

We analyzed refractive error, IOP, pRNFL thickness (Figure 2), mGCIPL thickness (Figure 3), cup-to-disc (CD) ratio, and macular thickness (Figure 4) differences between the two groups.

The paired* t*-test and the Wilcoxon signed-rank test were used to compare ocular parameters. All statistical analyses were performed using SPSS software for Windows, Version 21.0 (SPSS, Chicago, IL, USA). The statistical significance level was set at *P* < 0.05.

## 3. Results

### 3.1. Comparison of Normal Myopic Eyes and Myopic *β*-PPA Eyes

A total of 54 eyes of 27 patients [9 males (33%) and 18 females (67%)] met the inclusion criteria. The mean age was 25.33 ± 5.02 years.  Table 1 summarizes the demographics and baseline clinical characteristics. There were no statistically significant differences in IOP, corneal thickness, myopic error, astigmatism, or SE between myopic eyes without *β*-PPA and myopic eyes with *β*-PPA.

### 3.2. Macular GCIPL, Peripapillary RNFL Thicknesses, and ONH Parameters

Table 2 shows mGCIPL, pRNFL, and ONH parameters for the *β*-PPA and control eyes. The average of five out of six sectors and minimum values of mGCIPL thicknesses in eyes with prominent *β*-PPA discs were significantly less than those of the control eyes. The average pRNFL thickness in eyes with *β*-PPA was less than that in the control eyes, but with no significant difference in quadrant sector analyses. In clock-hour sector analyses, 6/6 sectors showed significant differences for pRNFL thickness. In ONH analysis, no significant difference was observed between myopic *β*-PPA and control eyes in the rim area, disc area, average CD ratio, vertical CD ratio, and disc volume.

Table 3 shows the average and the differences of the averages of macular thicknesses in nine sectors of the two groups. The macular thickness of the *β*-PPA eyes was thinner than control eyes in all sectors. There was a significant difference between the two groups in three sectors (the inner superior macula, inner temporal macula, and inner inferior macula), but there was no significant difference in the other sectors, including the fovea.

## 4. Discussion

This study showed differences of the macula and mGCIPL thicknesses between the myopic *β*-PPA and control eyes. The *β*-PPA is associated with myopic eyeball axial elongation and temporal pulling of the optic nerve. The adjacent retinal tissue extends externally, and this mechanical stretching results in a visible sclera [28–30]. A recent study reported myopic disc changes using serial optic disc photographs [14], and we assumed that the stretching forces on the retina included the macula and pRNFL thicknesses.

Although there was no significant difference in degree of myopia between the control and *β*-PPA eyes, *β*-PPA eyes had lower average values of mGCIPL thickness in five out of six sectors, compared with the control eyes. Previous studies of *β*-PPA and glaucoma used heterogeneous groups comprised of a wide variety with regard to race, ethnicity, age, and degree of myopia [18, 19, 31, 32]. There has been no study that reported possible associations of *β*-PPA with macular parameters.

The present study is therefore the first report to compare different ocular parameters between two eyes from the same person, to characterize associations of *β*-PPA with macular status. Using this approach, it was possible to determine associations between ocular parameters and myopic *β*-PPA, without other confounding factors.

A significant difference was evident in the mGCIPL between the two groups. It has already been established that mGCIPL thickness is a good indicator for early glaucoma detection, with excellent diagnostic performance in many studies [33–36]. Although a few studies reported some differences in mGCIPL thicknesses by ethnic groups [37, 38], there was little variation among our participants. However, because variations in macular structure with race and ethnicity are well known, more studies of mGCIPL thickness by race and ethnicity are needed [39–41]. The present study showed that the myopic eyes with *β*-PPA have a thinner mGCIPL than the myopic eyes without *β*-PPA. Only 6/6 sectors in clock-hour sector pRNFL analyses showed significant differences; the average and quadrant sector analysis of pRNFL and ONH analyses showed no significant differences. A recent study showed that the PPA developed toward the inferotemporal direction in 77.2% of myopia patients [42]. Although we did not group according to the direction of the PPA because of the small sample size, most common PPA directions were temporal or on the inferotemporal side with the reference line between the disc center and macula. This directional stretching may have affected the thicknesses of 6/6 sectors in the clock-hour sector of the pRNFL, but there were no significant differences in adjacent sectors of 6/6 clock-hour analyses or inferior quadrant analyses of the pRNFL. These results suggest that additional studies involving larger cohorts with close follow-ups are necessary to confirm and enlarge the results of the present study.

Recent studies have reported that the foveal thickness of myopic patients is thicker than that in emmetropia patients and increases with progression of myopia [43, 44]. Although the present study showed no significant differences of foveal thicknesses, because there was no significant difference in myopia, parafoveal retinal thickness was associated with *β*-PPA, which may be attributed in part to the difference in mGCIPL thicknesses. The difference in mGCIPL thickness was approximately 2–5 *μ*m, and the difference in macular thickness was approximately 4–6 *μ*m. We determined the inner retinal thickness at a distance of 0.5–1.5 mm from the foveal center, and the mGCIPL thickness was measured at a distance of 0.5–2.0 mm from the foveal center. The areas of these measurements therefore showed considerable overlap. A previous study reported that the average macular thickness of the foveal and parafoveal regions of myopic patients did not change with the degree of myopia, but the parafovea was thinner, and the fovea was thicker [45]. The present study also showed that myopia involving *β*-PPA is associated with a thinner inner macular thickness.

Before the use of OCT, it was thought that myopic changes mainly resulted from atrophy of the retinal pigment epithelium at the discs and posterior poles [46]. Recent studies using OCT have shown that the fovea is thicker in myopic eyes [43, 45]. Several studies have hypothesized that the increased axial length causes mechanical stretching of the sclera at the posterior pole. This stretching induces vitreal traction on the fovea, making it thicker [47, 48]. Another study suggested that foveal reconstruction by retinal stretching occurs in response to intraocular pressure and ocular growth in myopic eyes. As a result of foveal reconstruction, the parafovea, which is a more elastic tissue, becomes thinner [49]. In the present study, parafoveal thickness was thinner in myopic eyes, with a change in *β*-PPA eyes. Although there was no significant statistical difference, these eyes were more myopic and had a change in the *β*-PPA. However, no significant difference was observed in the foveal thickness between the *β*-PPA and control groups. This suggests that the initial change does not involve the fovea, and *β*-PPA arises from mechanical stretching of the retina by elongation of the eyeball. The thinning of the parafovea and mGCIPL occurring in myopic eyes with *β*-PPA suggests that this phenomenon may result from tangential mechanical stretching, and not from anteroposterior vitreous traction. Considering the results of the present study, foveal reconstruction is more reasonable, and parafoveal change may be an early sign of retinal change of the myopic eye.

As previously mentioned, several studies have reported that *β*-PPA develops by axial elongation [14, 28]. However, the current study involved the frequency of this process, and disc changes were not always accompanied by axial elongation, which varied among individuals. The patients included in this study also showed no significant differences in myopic error between the two eyes but they had different disc features. Furthermore, we showed that the myopic change of the disc reflected the myopic change of the retina, especially the parafovea.

There were some limitations in this study. We did not evaluate the axial length. Although there was no significant difference in myopic error between the two groups, verifying the axial length is required for accurate analyses with corrections using Littmann's method [50]. For the same reason, correlation analyses with the size of the PPA were not possible. The sample size was too small for subgroup analyses to determine the effect of *β*-PPA directions. As mentioned in Introduction, there are some recent studies which proposed a new classification for *β*-PPA using spectral-domain OCT image findings. They divided the *β*-PPA into newly defined *β*-PPA, an area with intact Bruch's membrane, and *γ*-PPA, an area devoid of Bruch's membrane. They suggested an association of *γ*-PPA and myopia [30, 51]. But until now, most studies have used classic definition of the *β*-PPA and this study also did not classify the *β*-PPA [19, 23, 24, 28].

In conclusion, when compared with myopic eyes without *β*-PPA, myopic *β*-PPA eyes show changes in mGCIPL and macular parameters. These changes can result merely from advanced myopic changes that cause impairment in visual function, or they can result from damages to the disc and retina, causing impairment in visual acuity and visual field. Additional studies with close follow-ups of these patients are therefore warranted. In addition, to better characterize correlations of myopic *β*-PPA, future studies should involve eyes with different directional *β*-PPA, diffuse *β*-PPA, and optic disc torsion.

## Figures and Tables

**Table 1:** Demographics and baseline clinical characteristics of the study participants.

	No *β*-PPA eyes	Myopic *β*-PPA eyes	*P* value
IOP (mmHg)	16.44 ± 3.41	16.14 ± 3.40	0.349
Central corneal thickness (*μ*m)	538.67 ± 32.88	538.74 ± 34.31	0.911
*Refractive error (diopters)*			
Myopia	−3.82 ± 1.60	−4.01 ± 1.61	0.109
Astigmatism	−0.93 ± 0.99	−0.83 ± 1.10	0.428
Spherical equivalent	−4.29 ± 1.83	−4.44 ± 1.83	0.229

IOP, intraocular pressure; *β*-PPA, *β*-zone parapapillary atrophy.

**Table 2:** Macular GCIPL, pRNFL thicknesses, and ONH parameters.

	No *β*-PPA eyes (control)	Myopic *β*-PPA eyes (case)	Difference (control-case)	*P* value
*mGCIPL (μm)*				
Average	81.07 ± 4.31	78.93 ± 4.18	2.15 ± 2.44	<0.001
Minimum	78.93 ± 4.72	73.85 ± 8.07	5.07 ± 8.95	0.007
Superotemporal	81.11 ± 5.18	78.33 ± 5.10	2.78 ± 3.94	0.001
Superior	82.07 ± 4.90	79.63 ± 5.23	2.44 ± 3.33	0.001
Superonasal	83.07 ± 5.36	80.93 ± 6.29	2.14 ± 5.34	0.047
Inferonasal	80.70 ± 4.56	79.30 ± 5.25	1.41 ± 4.82	0.141
Inferior	78.04 ± 4.89	75.89 ± 6.27	2.15 ± 5.34	0.038
Inferotemporal	82.33 ± 4.09	80.07 ± 5.01	2.26 ± 4.18	0.009
*pRNFL (μm)*				
Average	93.56 ± 8.51	91.44 ± 9.23	2.11 ± 4.42	0.020
Superior	117.70 ± 16.97	112.44 ± 20.83	5.26 ± 15.90	0.097
Temporal	74.33 ± 15.03	72.52 ± 13.55	1.81 ± 14.54	0.522
Inferior	118.93 ± 17.93	116.30 ± 15.16	2.62 ± 10.92	0.222
Nasal	64.07 ± 10.64	62.41 ± 9.78	1.67 ± 7.11	0.234
*Clock hours R/L*				
12/12	111.71 ± 27.19	105.63 ± 28.13	6.07 ± 20.92	0.130
1/11	109.85 ± 17.15	104.74 ± 21.54	5.11 ± 17.70	0.146
2/10	88.30 ± 17.20	84.63 ± 15.68	3.67 ± 17.73	0.293
3/9	55.00 ± 11.83	53.48 ± 11.14	1.52 ± 12.40	0.771
4/8	60.59 ± 8.71	60.37 ± 7.16	0.22 ± 7.56	0.880
5/7	92.33 ± 17.97	90.00 ± 16.11	2.33 ± 12.64	0.346
6/6	123.19 ± 26.93	115.81 ± 26.11	7.37 ± 16.39	0.007
7/5	146.00 ± 20.74	143.81 ± 17.91	2.19 ± 13.91	0.422
8/4	79.37 ± 10.66	79.15 ± 13.11	0.22 ± 10.86	0.916
9/3	55.37 ± 10.83	54.26 ± 10.48	1.11 ± 10.24	0.511
10/2	77.00 ± 10.88	78.44 ± 17.08	−1.44 ± 17.19	0.666
11/1	131.00 ± 15.36	132.19 ± 15.34	−1.19 ± 13.30	0.210
*ONH parameters*				
Rim area (mm^3^)	1.23 ± 0.17	1.20 ± 0.28	0.03 ± 0.21	0.416
Disc area (mm^3^)	1.74 ± 0.28	1.73 ± 0.31	0.01 ± 0.19	0.739
Average CDR	0.50 ± 0.13	0.50 ± 0.12	0.00 ± 0.08	0.829
Vertical CDR	0.46 ± 0.13	0.47 ± 0.12	−0.01 ± 0.08	0.611
Cup volume (mm^3^)	0.15 ± 0.12	0.15 ± 0.12	0.00 ± 0.09	0.983

mGCIPL, macular ganglion cell-inner plexiform layer; pRNFL, peripapillary retinal nerve fiber layer; ONH, optic nerve head; R, right; L, left; CDR, cup-to-disc ratio; *β*-PPA, *β*-zone parapapillary atrophy.

**Table 3:** Average of macular thickness (*μ*m).

	No *β*-PPA eyes (control)	Myopic *β*-PPA eyes (case)	Difference (control-case)	*P* value
Fovea	258.04 ± 16.10	255.52 ± 17.76	2.52 ± 8.68	0.143
Inner superior	322.85 ± 18.08	318.00 ± 16.37	4.85 ± 6.82	0.001
Inner temporal	309.22 ± 16.04	305.41 ± 15.57	3.81 ± 9.46	0.046
Inner inferior	318.15 ± 15.01	313.48 ± 12.91	4.66 ± 11.14	0.039
Inner nasal	326.41 ± 20.58	320.00 ± 14.97	6.41 ± 17.86	0.074
Outer superior	277.74 ± 13.30	276.63 ± 14.48	1.11 ± 5.42	0.080
Outer temporal	259.52 ± 14.62	258.89 ± 14.43	0.63 ± 10.79	0.764
Outer inferior	265.04 ± 12.30	264.67 ± 12.05	0.37 ± 8.63	0.825
Outer nasal	298.96 ± 14.72	294.96 ± 17.22	4.00 ± 12.72	0.114

*β*-PPA, *β*-zone parapapillary atrophy.
